# One size does not fit all: an application of stochastic modeling to estimating primary healthcare needs in Ethiopia at the sub-national level

**DOI:** 10.1186/s12913-023-10061-1

**Published:** 2023-10-06

**Authors:** Brittany L. Hagedorn, Rui Han, Kevin A. McCarthy

**Affiliations:** Institute for Disease Modeling, Bill & Melinda Gates Foundation, 500 5th Ave N, Seattle, WA 98109 USA

**Keywords:** Human resources for health, Workload, Ethiopia, Sub-national, Monte Carlo, Stochastic, PACE-HRH, Primary healthcare

## Abstract

**Background:**

Primary healthcare systems require adequate staffing to meet the needs of their local population. Guidelines typically use population ratio targets for healthcare workers, such as Ethiopia’s goal of two health extension workers for every five thousand people. However, fixed ratios do not reflect local demographics, fertility rates, disease burden (e.g., malaria endemicity), or trends in these values. Recognizing this, we set out to estimate the clinical workload to meet the primary healthcare needs in Ethiopia by region.

**Methods:**

We utilize the open-source R package PACE-HRH for our analysis, which is a stochastic Monte Carlo simulation model that estimates workload for a specified service package and population. Assumptions and data inputs for region-specific fertility, mortality, disease burden were drawn from literature, DHS, and WorldPop. We project workload until 2035 for seven regions and two charted cities of Ethiopia.

**Results:**

All regions and charted cities are expected to experience increased workload between 2021 and 2035 for a starting catchment of five thousand people. The expected (mean) annual clinical workload varied from 2,930 h (Addis) to 3,752 h (Gambela) and increased by 19–28% over fifteen years. This results from a decline in per capita workload (due to declines in fertility and infectious diseases), overpowered by total population growth. Pregnancy, non-communicable diseases, sick child care, and nutrition remain the largest service categories, but their priority shifts substantially in some regions by 2035. Sensitivity analysis shows that fertility assumptions have major implications for workload. We incorporate seasonality and estimate monthly variation of up to 8.9% (Somali), though most services with high variability are declining.

**Conclusions:**

Regional variation in demographics, fertility, seasonality, and disease trends all affect the workload estimates. This results in differences in expected clinical workload, the level of uncertainty in those estimates, and relative priorities between service categories. By showing these differences, we demonstrate the inadequacy of a fixed population ratio for staffing allocation. Policy-makers and regulators need to consider these factors in designing their healthcare systems, or they risk sub-optimally allocating workforce and creating inequitable access to care.

**Supplementary Information:**

The online version contains supplementary material available at 10.1186/s12913-023-10061-1.

## Introduction

The design of healthcare systems is variable across countries depending on the local context, affected by the resources available, health priorities, and cultural preferences. To meet the needs of a population, primary healthcare systems require a properly staffed healthcare workforce [1]. Building a fit-for-purpose workforce requires planning, training, certification, placement, and continuing education, as outlined by the World Health Organization (WHO) in their six building blocks for primary healthcare framework [2].

To estimate the number of trained workers that are needed, primary healthcare systems typically use health-workers-to-population ratio-based targets. For example, the WHO established a global threshold of 4.5 doctors and nurses/midwives per 1,000 population to achieve human resources for health goals of 80% coverage across 12 health indicators. The indicators selected are linked to the health sustainable development goals, which are based on empirical analysis of historical trends across specific geographies [2]. This is an update from the previous threshold of 2.3 physicians and nurses/midwives per 1,000 population identified in the 2006 World Health Report [3] to attain 80% coverage of skilled birth attendance. As demonstrated by these updates, static values are inadequate to account for evolving health priorities, coverage targets, and disease burden.

These ratios have been useful for aggregate planning [4, 5], but since they are globally derived values, they do not reflect the differences in demographics, socio-economic access to care, or disease burden between geographies and populations. Recognizing the need for more tailored staffing models, many countries have established similar, population ratio-based targets. For example, India set a target ratio of one Accredited Social Health Activist (ASHA) per 1,000 population [6] and Nigeria established a goal of one primary healthcare center (PHC) per ward of 10,000 to 30,000 people [7]. The methods by which these national standards are set are not often well-documented in the literature, but even if they originally came from a bottom-up process of estimating workload and the needed number of healthcare workers, once the ratios are enshrined as targets, the flexibility to adapt them locally or update based on trends over time stagnates.

As a result, these targets still face the challenges of being static in time, not being specific to the needs of sub-populations, and not reflecting the practicalities of local delivery systems such as population density. In addition, the rate at which disease burden and fertility are changing are heterogeneous at a sub-national level [8], so the geographical and temporal variation in healthcare needs are not reflected in a fixed ratio.

Even with a constant package of services, local incidence and demography impact the necessary healthcare worker capacity. Given this context, we set out to quantify the magnitude of sub-national and temporal variation in primary healthcare workload faced by healthcare workers to understand the implications of fixed-ratio planning. In doing so, we question whether fixed staffing ratios are adequate to ensure that a healthcare system can meet a population’s dynamic health needs.

Ethiopia’s health extension program (HEP) has been widely viewed as successful and the government has recently completed a planning process for the program over the next fifteen years [9], making it an interesting potential case study. We use this as the basis for assessing the primary healthcare workload across multiple regions of the country, projecting forward from 2020 to 2035 to align with their strategic roadmap.

## Methods

We use a previously published, bottom-up time accounting approach [10] to estimate the level of workload for each primary healthcare service included in a comprehensive service package. The list of healthcare tasks (“services”) is based on the Ethiopian HEP optimization roadmap for 2021–2035 [9] (complete list in Additional file 1, Table S1). This is a general primary healthcare service package with a broad range of services including immunization, antenatal care, family planning, nutrition, testing and treatment for both infectious and non-communicable diseases (NCDs), and routine preventive care and counseling. The analysis is run in R, based on the publicly available PACE-HRH software package [11].

### Model structure

The detailed model structure is outlined in a different publication [10] and we summarize it here. The model calculates the total amount of direct clinical contact time (in minutes) required to meet all of the healthcare needs for a baseline population. We start with a population of 5,000 in 2020, which is the target population size that each primary healthcare facility (Health Post) is supposed to serve [9], although the actual catchment population varies depending on spatial distribution, varying fertility, and internal migration.

For each service, we break out the relevant tasks (e.g. testing, treatment, counseling), specify the relevant population to which this service applies (e.g. children under 5, pregnant women), the number of contacts with the health system to fulfill the service (e.g. 1 test per suspected malaria case), the amount of time to complete the task, and lastly, the incidence or prevalence rate to reflect the proportion of the relevant population in need of care. The relevant population and the incidence rate are used to calculate the number of people expected to receive the service each year, which is multiplied by number of contacts and the time per contact to get the total service time for each PHC service. 

$$Total\,Service\,Time= \sum\limits_{all\,services}(Relevant\,population\,*\, Incidence\,rate\,*\,Number\,of\,contacts\,*\, Minutes\,per\,contact)$$

Administrative tasks, in-service trainings, community engagement, public health surveillance, travel, and related tasks can be optionally included in the model. For this study, we have chosen to focus on the population-based drivers of workload, so only include direct clinical care tasks.

We utilize a Monte Carlo simulation to estimate the expected time required for each task, and then sum all tasks to calculate the total clinical workload. The model includes both parameter uncertainty (the initial rate) and the stochasticity (year-over-year change) inherent in estimating future trends. The sampling occurs for parameters including fertility and mortality rates by five-year age groups, disease incidence rates, and time per contact (details of distributions in Additional file 1, Table S2).

We account for birth seasonality and time-varying conditions including malnutrition, tuberculosis (TB), malaria, and diarrhea by applying condition-specific seasonality curves to each relevant service. For example, birth seasonality is applied to antenatal care (ANC) visits, which requires 4 contacts with the health system. The seasonality curves are detailed in Additional file 1, Table S3 and offsets in in Additional file 1, Table S4.

To demonstrate the calculation method, Table 1 lists data inputs required by the model to compute total expected service time for a clinical task, using treatment for moderate acute malnutrition in under-five children as an example.

**Table 1:** Input parameters for task time calculations. Every clinical task in the model requires a set of inputs that are used in the time calculation. One example, for Amhara region, the task of treating moderate acute malnutrition in under-five children, is shown here. Incidence rate or prevalence rate are used as appropriate for the task being described. Annual change rates are calculated as a ratio to the prior year (i.e., 1.0 = no change)

Task time input parameter	Example value
Name of task	Treatment for moderate acute malnutrition
Relevant population	Children aged 0 to 59 months
Incidence/prevalence rate in the population	41.5% of under-five children
Number of contacts with the health system	2 contacts for malnutrition treatment
Time per contact with the health system	5 min per contact
Annual change in incidence/prevalence rate	0.98
Applicable seasonality curve	Malnutrition
Seasonality offset (months)	0 for the first contact, + 1 for the second contact

### Data collection and assumptions

We collected data for subnational modeling from a range of sources, depending on availability and quality. The model accounts for differences between regions across each of the key input variables described in Table 1 and uses the most localized data possible.

We utilized WorldPop population estimates adjusted to United Nations Population Division (UNPD) estimates [12] for regions of Ethiopia by 5-year age bands, interpolated to 1-year cohorts and modeled future population pyramids based on trends in fertility and mortality. Fertility rates are based on DHS-reported regional fertility rates from 2019 [13]. Mortality rates are sourced from the World Development Indicators (WDI) from 2020 [14]. We calculated the annualized rate of year-over-year change from the most recent pair of consecutive observations. In the cases where recent trends conflict with long-term historical patterns or when we were uncertain about whether a rapid change can persist, we include additional historical data in calculating the annualized change rate to get a more stable trend.

We included seasonal variation in workload for births [15] (for pregnancy-related services and immunization), diarrhea [16], malnutrition [17], malaria [18], and TB [19]. No other services are known to be highly seasonal, so demand for the remaining services are assumed to be evenly distributed throughout the year.

We searched Google and Google Scholar for published research articles, national prevalence surveys, and surveillance surveys to gather data on regional disease incidence rates, annual change rates in disease incidence, and regional seasonality patterns for disease incidence. In cases where regional data were not published, but the disease is known to exist, we backfilled with national averages, based on the expectation that the national average is the next-best estimate for the region. Where the data are inconsistent, we use the most recent available and prioritize estimates found in population-based surveys and meta-analyses with random sampling techniques. Due to gaps in the literature, it was difficult to find reliable estimates for annual change rates in regional disease incidence. Accounting for local disease eradication efforts and long-term trends in disease prevalence, we assumed a moderate decline of two percent per year for the relevant infectious diseases. Assumptions for number of contacts are based on common practice, and minutes per contact are educated guesses, which were also validated by experts.

Detailed data sources for all model inputs are listed in Table 2. Parameter values for each region are available in Additional file 2.

**Table 2:** Primary model inputs and data sources. Input parameters for the model by category are listed with their data source, year of data collection, applicability to various regions, and known limitations. The metrics were chosen to best capture the expected healthcare needs of relevant population for each service category. The most recent and the most localized data are used depending on data availability and quality. Where region-specific data was not available, neighboring regions or national averages were used instead in the model parameterization

Category	Source	Metric	Year	Coverage	Limitations
FP	DHS [20]	Total demand for FP, ages 15–49	2016	All regions	
FP	DHS [20]	Utilization of contraceptives	2016	All regions	
FP	DHS [20]	Rate of switching methods in last 12 months	2016	National	
FP	National sample [21]	Abortion per 100 pregnancies	2014	All regions	Legal status has evolved and may affect rates
Nutrition	Meta-analysis [22]	Treatment rate for malnourished mothers	2019	National	
Nutrition	DHS [20]	Severe malnutrition in children under 5	2019	All regions	Malnutrition is highly variable
Nutrition	DHS [20]	Moderate malnutrition in children under 5	2019	All regions	Malnutrition is highly variable
Nutrition	DHS [20]	Severe anemia in children under 5	2016	All regions	
Nutrition	DHS [20]	Severe anemia in children ages 5–18	2016	National	
Nutrition	DHS [20]	Severe anemia in adults	2016	All regions	
Pregnancy	National survey [23]	Emergency obstetrics	2015	Some regions	Incomplete representation
Pregnancy	Meta-analysis [24]	Maternal sepsis	2014–2019	Some regions	Regional data time points vary
Pregnancy	Observational study [25]	Mastitis	1998	USA	Data is old and not Ethiopian
Pregnancy	Meta-analysis [26]	Postpartum hemorrhage	2021	All regions	Regional data time points vary
Sick child	DHS [20]	Pneumonia in children under 5	2016	All regions	Poor data for ages 5 +
Sick child	DHS [20]	Diarrhea in children under 5	2016	All regions	Poor data for ages 5 +
Sick child	Meta-analysis [27]	Parasites in primary school students	2020		May not be representative
Sick child	DHS [20]	Fever excluding diarrhea and ARI in children under 5	2016	All regions	Poor data for ages 5 +
NCDs	Community survey [28]	Age-specific hypertension	2015	National	May not be representative
NCDs	Community survey [28]	Age-specific diabetes	2015	National	May not be representative
NCDs	WHO estimates [29]	Age-specific cancer	2020	National	Cancer is likely under-reported
NCDs	Meta-analysis [30]	Asthma	2020	National	Asthma is likely under-reported
NCDs	Global model [31]	Ophthalmic disease in children ages 0–9	2013	National	Not direct observation
NCDs	Community survey [32]	Ophthalmic disease in adults	2020	National	Indirect measurement
Malaria	Surveillance data [33] & modeled estimates [34]	Malaria cases in children and adults	2009, 2020	Regional, National	Likely under-reported in surveillance
NTDs	Surveillance survey [35]	Scabies	2020	Regional	Outbreak setting
NTDs	Cross-sectional survey [36]	LF	2015	Nine regions	
NTDs	Prevalence surveys [37–39]	Trachoma	2008–2019	Regional	MDA may have reduced incidence
NTDs	Meta-analysis [40]	Soil-transmitted helminths in children	2000–2019	National + some regions	Limited reporting affects data quality
Sexual health	DHS [20]	Incidence of STIs among men & women 15–49	2016	All regions	Metrics are coarse
Sexual health	DHS [20]	Sexual and physical violence	2016	All regions	Susceptible to underreporting
First aid	DHS [20]	% of households with injury lasting < 7 days, 8–30 days	2016	All regions	Susceptible to underreporting
TB	Surveillance-derived model [41]	Incidence of TB	2017	All regions	
HIV	UNAIDS estimates [42]	Incidence per 1,000 uninfected adults	2020	National	
HIV	DHS [20]	Prevalence, women ages 15–49	2020	All regions	Regional data quality varies
HIV	UNAIDS estimates [43]	Prevalence, all adults ages 15–49	2020	National	
Mental health	National health survey [44]	Prevalence of depression	2003	National	Old survey & susceptible to underreporting
Fertility rates	DHS [20]	Age-specific fertility rates (5-year bands)	2019	All regions	Sample size limitations
Mortality rates	DHS [20]	Infant mortality rate, child mortality rate	2019	All regions	Sample size limitations
Mortality rates	WDI [45]	Mortality ages 5–9, 10–14, 15–19, 20–24, adult female & male	2021	National	

*FP* family planning, *DHS* demographic and health survey, *BMI* body mass index, *NCD* non-communicable disease, *WDI* world development indicators, *TB* tuberculosis, *NTD* neglected tropical disease, *LF* lymphatic filariasis, *HIV* human immunodeficiency virus, *STI* sexually transmitted infection, *MDA* mass drug administration, *ARI* acute respiratory infection, *WHO* World Health Organization, *UNAIDS* United Nations AIDS organization

### Study setting

We forecast PHC clinical workloads at the regional level from 2021 to 2035. As of 2023, Ethiopia is composed of eleven National Regional States (regions) – Tigray, Afar, Amhara, Oromia, Somali, Benishangul-Gumuz, Southern Nations, Nationalities and Peoples’ Region (SNNPR), Sidama, South West Ethiopia Peoples’ Region, Gambela, and Harari – and two chartered cities – Addis Ababa and Dire Dawa. The Sidama region and the South West Ethiopia Peoples’ Region were split off from the SNNPR in 2019 and 2021 respectively. Due to the recency of these changes and the age of our data sources, we treat the Sidama region and the South West Ethiopia Peoples’ Region as part of SNNPR for expediency purposes; this should not be construed as an opinion on the changes to administrative borders.

We exclude Tigray and Afar in the analyses because Tigray and parts of Afar were substantially affected by civil conflict from November 2020 to November 2022 [46, 47], making the reliability of disease burden estimates questionable since historical trends likely no longer hold and some infectious diseases and malnutrition may have had a resurgence due to low service coverage during the conflict. Additionally, prior to the onset of conflict, Afar had the highest overall fertility rates in the country and the fertility rates were increasing for the youngest age groups. The conflict could affect the age-group specific fertility and mortality rates in ways that are hard to predict, so making future estimates is potentially risky if they are used by decision makers, until the situation has fully stabilized and new data has been gathered.

### Model application

We ran the model under two specifications. The first specification projects forward population growth based on historical trends in fertility [48]. The second specification holds a fixed population of 5,000 for the duration of the simulation, which is the stated target population ratio for a PHC center in Ethiopia and represents the scenario if the country met and continued to achieve its access goals. Comparing the two specifications isolates the expected changes in clinical workload due to population structure shifts only. The analyses are based on simulated results from 100 trials for each model specification.

Even though fertility rate predictions are a key driver of population size in the model, it is uncertain whether future trends will mirror the recent history on which we have based our model parameters. Rates are known to change in response to a complex set of dynamics, including social norms, access to family planning, and girls’ education, and have been known to evolve in unexpected ways [49]. To understand the impact of this uncertainty about the future, we conducted sensitivity analysis to assess its impact on the predicted PHC workload. We reran the model using two different historical time periods for our parameters: 2011–19 and 2000–19, and a benchmark of moderate decline in fertility rates at 0.5% per year relative to baseline.

We use two settings: Addis Ababa and Oromia as comparators. Addis Ababa, the capital city, has a different fertility pattern than the rest of Ethiopia and the degree to which rates are changing has varied in recent years. Addis had the lowest current fertility rates in the country as of 2019 (e.g., 0.1 for women ages 20–24 [13]), but they rose in all age groups from 2000 to 2019 [15]. In contrast, Oromia has fertility rates significantly higher than Addis Ababa as of 2019 (e.g., 0.235 for women ages 20–24), but they decline in all age groups from 2000 to 2019. Oromia exhibits larger declines in fertility rates during the period 2011–2019 [15]. To test the impact of the fertility assumptions on model results, we reran the model with different plausible fertility trajectories for Addis Ababa and Oromia as comparators.

The analyses were completed using PACE-HRH [11] release version 1.0.2. The model was built in R Studio version 2022.07.2 + 576, using the R statistical programming language version 4.2.0. Code is publicly available on GitHub.

## Results

### Regional variation in clinical workload

**Fig. 1:**
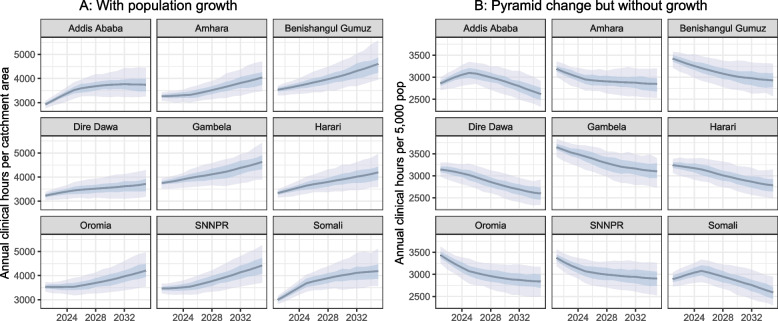
Predicted clinical workload, by region. The solid line in the center of each plot represents the median value of 100 simulation trials, for the annual hours of direct clinical care time required to meet the needs of a region’s population, from 2021 to 2035. Does not include any non-clinical time such as training, outreach and education, or supply management. Shadings represent the 25th and 75th (dark shading), and 5th and 95th (light shading) percentiles of simulated results. Panel **A** Estimates are for a catchment area population of 5,000 in 2020 that increases with population growth over the 15 years modeled. Panel **B** Estimates are for a fixed population of 5,000 individuals, with an evolving population pyramid (based on local demographics, fertility, and mortality), but without total population growth

With population growth, the total clinical workload is predicted to rise in all regions in the 15 years from 2021 to 2035. Of the regions modeled, Addis Ababa had the lowest total clinical workload in 2021 at 2,930 h per health post per year, increasing to 3,742 h by 2035 (61–78 h per week, assuming 48 workweeks per year). Gambela had the highest workload, increasing from 3,752 to 4,626 h per health post per year. The gap between the regions with the highest and the lowest predicted clinical workload expands from 17 h per week in 2021 to 19 h per week in 2035 (28% and 25% of the lowest regional workload, respectively) (Fig. 1, Panel A).

Across the regions, there are three general shapes to the workload curves, which reflect the combined effects of population growth and shifts in the population pyramid, partially mitigated by the anticipated decline in infectious diseases.

- Addis Ababa displayed the most pronounced slowdown and workload was projected to stabilize by 2028.
- Dire Dawa and Somali have decelerating growth rates and began to flatten in later years.
- In the remaining regions, the workload predictions for Gambela, Harari, Oromia, and SNNPR rise at a modest rate in the first few years and then saw an acceleration of the growth in workload by 2035.

While the total increased, the workload *on a per-capita basis* is projected to decline in all regions. (Fig. 1 Panel B) Within these per-capita declines, most regions decline consistently, except for Addis Ababa and Somali. In those two regions, the per-capita workload is predicted to rise initially in the first few years before eventually starting to decline. This reflects the combined effect of lower proportions of children under age 5 in year 2020 (8% in Addis, 11% in Somali, near 15% in other regions) and fertility rates that are either comparatively high or growing more rapidly, thus resulting in a near-term baby boom (Additional file 1, Table S1).

Overall, both the total level clinical workload and the shape of the growth curve varied by region. We explored the relative impact of the contributors to these differences, such as regional disease burden, initial demographics, and fertility rates, in the following sections.

### Primary clinical service categories

**Fig. 2:**
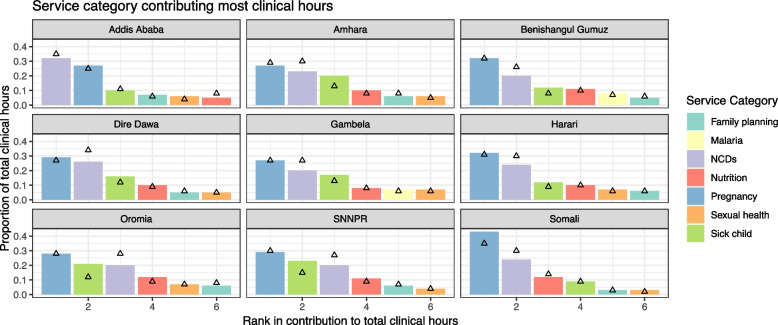
Breakdown of service categories as a percentage of the total clinical hours. Values are the median of the percentage calculations from 100 simulation trials. Bars represent values for 2021. Triangles represent values for 2035. The breakdown in 2021 reflects the current state of contributions to total clinical needs by each service category based on existing evidence of disease incidence rates and demographic characteristics of each region. The breakdown in 2035 reflects both the predicted change in demographics and the expected trend in local disease burden based on development observed in the past decades. Pregnancy related services include antenatal care, delivery, childbirth complications, postnatal care, and newborn care. Sexual health includes treatment of menstrual problems, response to and medical treatment for gender-based violence, and sexually transmitted infections management. Sick child care includes case management of fever, diarrhea, parasites, scabies, and pediatric palliative care in children under five. NCDs include screening and care for non-communicable diseases, including hypertension, diabetes, cancer, asthma, and ophthalmic diseases. The service categories not ranked in the top six in any region are not displayed, including first aid, HIV, mental health, neglected tropical diseases, and tuberculosis

To understand which services contributed most to the total clinical workload, we calculated the proportional contribution to the total by service category (a total of 12 categories, Table 2), by year (Fig. 2). We find that pregnancy, NCDs and sick child care are the top three contributors in 2021 (except for in Somali) and remain in the top four in 2035. Some categories only appear in the top contributors for certain regions, such as malaria, or substantially vary in magnitude, such as malnutrition.

The proportional contribution by service category changes over time based on expected trends in demographics and disease burden, resulting in shifts in the ranking. In Benishangul-Gumuz and Harari, malnutrition overtakes sick child care to become the third largest concern by 2035, primarily due to expected decline in incidence of childhood disease. Providing care for sick children was a large proportion of the workloads in Oromia and SNNPR in 2021, but its contribution dropped by a third by 2035. NCD-related workload rose in all regions, especially for Oromia and Dire Dawa, overtaking sick child and pregnancy-related work in some regions.

### Impact of fertility trend assumptions

**Fig. 3:**
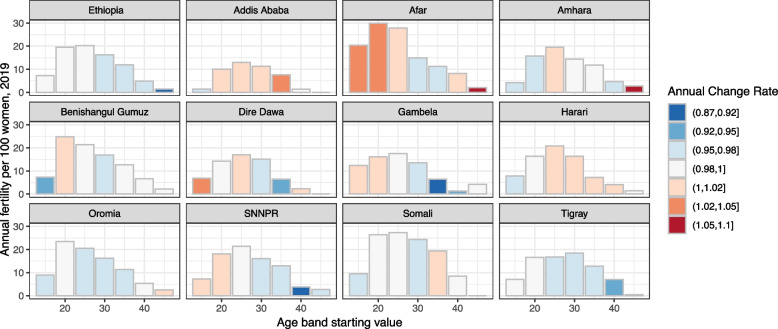
Annual fertility per 100 women, DHS 2019. Bars show the fertility rates by 5-year age band reported in the DHS 2019, by region. Shading indicates the rate at which those baseline values are changing. Annualized rates of year-over-year change are calculated from DHS 2019 and DHS 2011 for all regions except for Addis Ababa. DHS 2019 and DHS 2000 are used to calculate year-over-year change for Addis Ababa

Fertility rates underpin the predictions for future population, and thus have a large impact on the results of the prediction model. At the national level, fertility rates are declining for all age groups, but this is not the case for individual regions. (Fig. 3) Pastoral regions (e.g., Afar, Benishangul-Gumuz, and Somali) have the highest fertility rates. Addis Ababa, Afar, and Harari stand out for having an increasing trend in fertility rates for the most productive age groups.

**Fig. 4:**
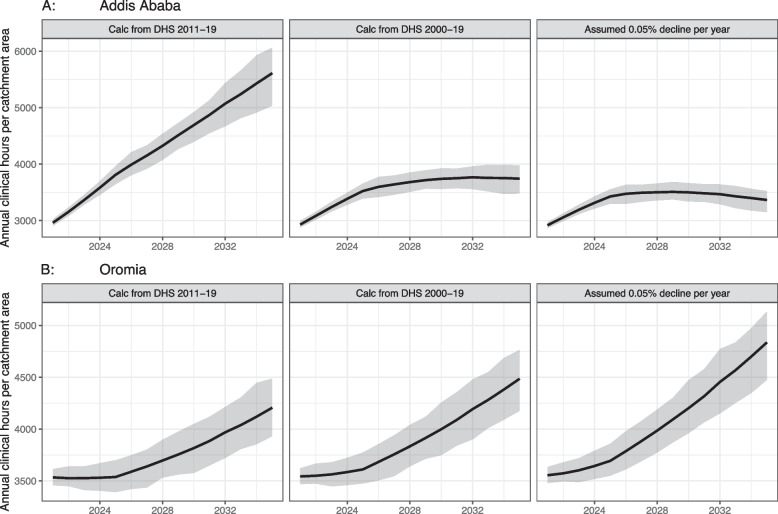
Total annual hours of direct clinical care for Addis Ababa (Panel **A**) and Oromia (Panel **B**), under different fertility assumptions. The first two panels show estimates based on annual growth rates in fertility calculated across different time points in DHS. The third panel shows estimates based on an assumed low-level 0.05% decline per year for comparison purposes. The solid lines in the center of each panel represent median of predicted workload, and the shaded areas represent a 50% simulation interval. Estimates are for a catchment area population of 5,000 in 2020 that increases with population growth over the 15 years modeled. Does not include any non-clinical time such as training, outreach and education, or supply management

Ethiopian Public Health Institute (EPHI) [Ethiopia], ICF [13] and USAID Demographic and Health Surveys Program [15]. We find that assumptions on fertility rates greatly affect our estimates of clinical workload in Addis, but less so in Oromia. If future trends correspond to those in the last 10 years, the total workload in Addis will be 50% higher by 2035 than if the trends correspond to the longer-term 20-year historical trend. The corresponding difference is 6% lower for Oromia (Fig. 4).

### Magnitude of seasonal variability in workload

**Fig. 5:**
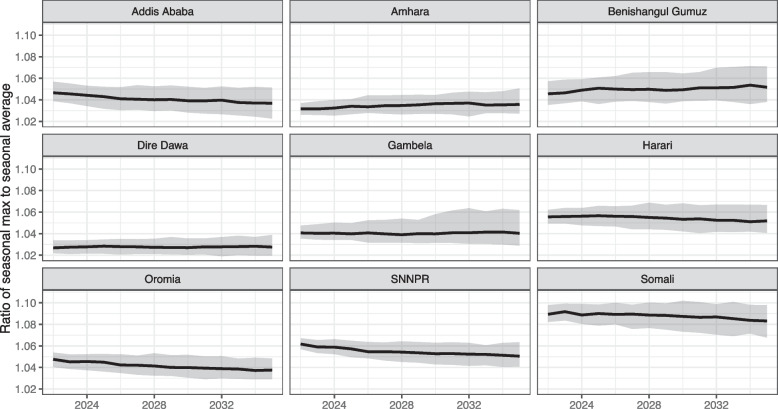
Ratio of clinical workload for the peak month to the average monthly clinical workload, by region. The peak month is the month predicted with the highest clinical workload of each year. The solid lines indicate the median value from 100 simulation trials, and the shaded areas represent a 90% simulation interval

To assess the impact of seasonality, [15–19] we calculated the ratio of the highest month (maximum workload) to the average value. Figure 5 shows some regions should expect more variability than others; Somali had the most seasonal variation with the peak month 8.9% (90% CI 8.2%, 9.8%) above average in 2022. Most regions have declining demand for services affected by seasonality, which results in a slow decline in the calculated ratio. Additional file 1, Figure S2 displays variation in clinical workload by month of the year for each region.

### Clinical hours for age-specific population

**Fig. 6:**
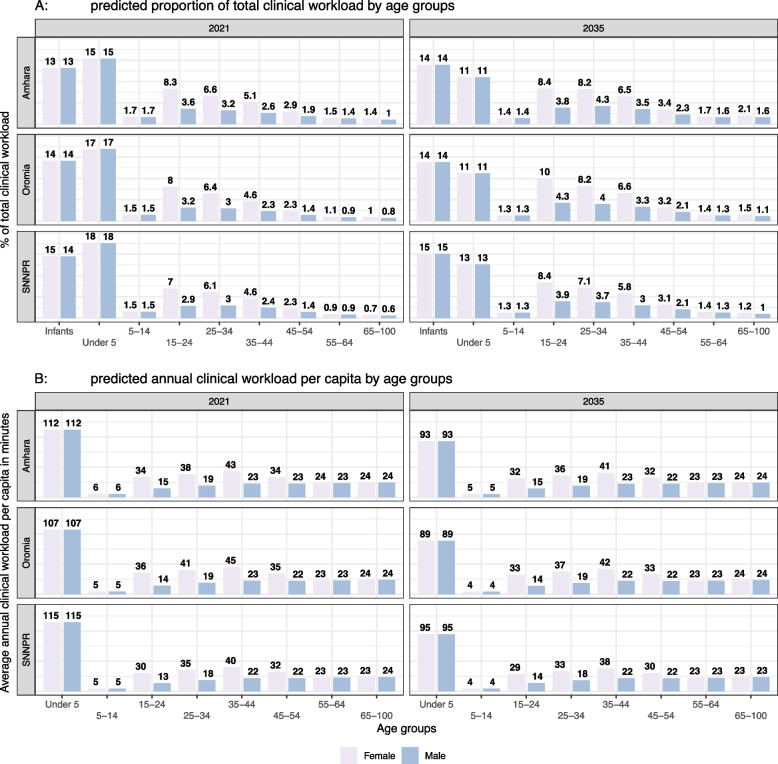
Predicted clinical workload based on age group classifications, for Amhara, Oromia, and SNNPR. Does not include any non-clinical time such as training, outreach and education, or supply management. Values are the mean of calculations from 50 simulation trials. Population between the age of 0 and 100 are assigned into following groups: infants (i.e., age under 1), under 5 (i.e., age of 1 to 4), 5–14, 15–24, 25–34, 35–44, 45–54, 55–64, 65–100. Panel **A** Proportion of total clinical workload contributed by each age group, female, and male. Panel **B** Average annual clinical workload per capita in minutes, by age groups, female, and male

We examined clinical workload for age-specific population in the three most populous regions of Ethiopia: Oromia, Amhara, and SNNPR. For all three regions, infants and children under the age of 5 years are the groups predicted to accrue the highest proportion of clinical workload, accounting for a combined 57.2% of predicted clinical workload in Amhara, 62% in Oromia, and 64.5% in SNNPR (Fig. 6, Panel A). The regions show slight differences in proportions of workload accrued by different age groups due to demographics and disease burden.

From 2021 to 2035, the per capita workload for children under 5 are predicted to decline due to expected decrease in incidence of childhood diseases (Fig. 6, Panel B). During the reproductive ages from 15 to 49, the per capita workload is higher for female compared to male, explained by utilization of family planning services by females.

This analysis focused on primary healthcare in a low-income setting, which constrained the inclusion of clinical workload on the treatment of some leading causes of morbidity and mortality in population aged 65 and over (e.g. Alzheimer’s care, cancer treatments, and liver disease treatments).

## Discussion

In summary, we find that the total annual primary healthcare clinical workload varies by region by up to 28%, due to differences in population pyramid, fertility, and disease incidence. This will shift over time in regionally specific ways as demographics and disease burden evolve, with some regions stabilizing at a higher-than-current workload over 15 years, while others will continue to rise rapidly.

On net, our estimates show that for a given population in 2020, the workload will rise substantially across all regions by 2035. The increase in workload is predominantly due to the growing population (estimated to rise by 40% between 2020 and 2035, per UN estimates [48]).

The clinical workload is projected to decline on a per-capita basis because of the combined effect of the young population aging into their healthiest years and expected reductions in disease rates. However, this is dependent on continued declines in net fertility rates and decreasing levels of infectious diseases, which are not guaranteed to materialize.

The top three service categories were pregnancy-related services, non-communicable diseases, and sick child care, with sexual health, malnutrition and malaria being important as well in region-specific ways. Local disease burden and demographics influence the variability of each service category’s rank across regions. For example, malaria ranks in the top six in the two regions [50] with the highest malaria incidence. Malnutrition is a higher priority concern in Somali due to food insecurity, compounded by a young age pyramid and high fertility.

Several of these categories are affected by seasonal variability and planning based on average monthly clinical workload would underestimate staffing need by up to 10%. Underestimation for workload due to seasonality is more severe in regions with higher fertility rates (Somali) and regions with higher burdens in conditions subjected to seasonality (Benishangul-Gumuz, Somali, and SNNPR). Given this level of variation, we conclude that seasonality is an important element to consider during the healthcare workforce planning process, even if not all PHC services display a seasonal pattern.

Based on these analyses, we make three key observations. First, the regional PHC needs are closely linked to the local context of disease incidence rates and demographic characteristics and the combined effects of both contribute to non-trivial cross-region differences in both amounts of clinical time required and areas of attention. Second, the regional differences do not dissipate over time and attention needs to be paid to both the shared trends (e.g., the increasing burden in NCDs), and the localized trends (e.g., the increasing burden in malnutrition for Benishangul-Gumuz and Harari). Third, even if a declining per-capita workload does materialize, the population growth may make it difficult for the country to ‘keep up with’ demand for services.

As far as we are aware, this is the first application of a stochastic workload model to estimate health workforce needs at a sub-national level. Previous studies have used static models to project health worker needs, mostly at the national level [51–53], with a few sub-national applications [54–56]. However, none have previously incorporated stochastic population trajectories, uncertainty in disease incidence, or seasonality, as far as we have uncovered in the literature. Additionally, no comprehensive sub-national modeling has been previously done in Ethiopia.

As with any study, there are limitations. Primary here is that this exercise was intended to examine future trend and regional differences, but numeric values should be used with caution due to limited data availability, especially the lack of time and motion studies that reported data at the clinical tasks level. Additionally, data on regional disease incidence rates was lacking for health areas that have not historically been prioritized by Ethiopia’s health sector [57] or that go under-reported, such as leishmaniasis, cancer treatment, gender-based violence, and smoking cessation. Another challenge is projecting trends, especially for fertility and disease incidence, over the fifteen-year modeled time horizon. However, while future trends are never certain, this should not limit us from anticipating expected changes and planning ahead to meet those needs.

Future work is needed to refine and expand upon these results. First is that improved data on incidence rates, either through new collection or by making use of local surveillance records, would substantially improve the reliability of estimates for local planning. Second is the need to consider anticipated fertility trends and update models routinely as the future unfolds. It is important to have accurate census data on populations and the modeling points to the potential use of admin data on family planning use to track changes in more real-time. Third is that this modeling estimates needed capacity in order to inform capacity planning, but it does not estimate the health or operational consequences when there is inadequate capacity available. For example, modeling could be used to estimate user waiting times, impact on care seeking rates, health worker burnout risk, and ultimately the health impact of missed opportunities to provide health services.

## Conclusion

There are a few recommendations for policy makers who would like to learn from this study. Based on these analyses, we conclude that the regional variation in workload, which compounds over time, means that staffing equally based on the national average would result in inequitable access to care. These regional differences should be considered when allocating resources for training and recruitment of healthcare workers, both in Ethiopia and in other countries with similarly diverse populations. Additionally, national policies should incorporate allowance for flexible staffing models that are tailored to local needs.

Staffing shortfalls may be compounded by seasonality, leading to a reduction in access to care during peak months. One option for addressing this in resource-constrained settings is for tasks that are less time sensitive (e.g., community health education) to be shifted to lower-workload times of year (e.g., malaria off-season) in a strategic way to reduce the impact of seasonality on overwhelmed health workers.

We also conclude that while normative staffing ratios are useful for aggregated goal setting, they are inadequate for localized planning because they do not adequately consider the contextual differences in health needs, and how those evolve over time. Thus, health systems would be better able to ensure adequate resourcing if planning is based on population-aware workload projections.

### Supplementary Information

**Additional file 1:** **Table S1.** List of Health Extension Program (HEP) primary health care services(25), grouped in service packages. These services are expected to be delivered at the health posts. **Table S2.** Probability distribution specifications for stochastic parameters. Random sampling occurs for the listed model parameters based on the chosen probability distribution. Mean and delta values for each model parameter are sourced from reported data. Values for p and q are chosen to reflect the desired range of values to sample from. **Table S3.** Seasonality Curves. Each column of the table represents one seasonality curve, reflecting the relative frequency of observing the condition in each month of the year. The sum across all months of the year for each curve is 1. **Table S4.** Seasonality Offsets. Seasonality curves are applied to relevant clinical tasks based on the listed offset values for each contact. An offset value specifies the number of months a seasonality curve needs to shift to match the timing of the contact with the health system to receive service: 0 for as is, a positive value for shifting forward, and a negative value for shifting backward.  **Figure S1.** Population composition in 2020 by age group and region. The total population is shown, broken out by age group, gender, and region. The population includes individuals between the age of 0 and 100, assigned into following groups: infants (i.e., age under 1), under 5 (i.e., age of 1 to 4), 5-14, 15-24, 25-34, 35-44, 45-54, 55-64, 65-100. **Figure S2.** Seasonality of clinical workload. The month-by-month variation of the clinical workload is shown, broken out for each region, in 2022 and 2035. Values are the ratio of predicted clinical workload for the month, relative to the average predicted monthly workload of the year. Shown are the average values calculated from 100 simulation trials.

**Additional file 2.**

## Data Availability

All code used to run the models is publicly available on GitHub at https://institutefordiseasemodeling.github.io/PACE-HRH/ and model input and results csv files are available upon request.

## Abbreviations

WHO: World Health Organization
HRH: Human Resources for Health
PACE-HRH: Population-aware capacity estimator for human resources for health
ASHA: Accredited Social Health Activist
PHC: Primary Healthcare Center
HEP: Health Extension Program
NCDs: Non-Communicable Diseases
ANC: Antenatal Care
TB: Tuberculosis
UN: United Nations
WDI: World Development Indicators
SNNPR: Southern Nations, Nationalities and Peoples’ Region
DHS: Demographics and Health Surveys
HIV: Human Immunodeficiency Virus
BMGF: Bill & Melinda Gates Foundation
FP: Family Planning
BMI: Body Mass Index
NTD: Neglected Tropical Disease
LF: Lymphatic Filariasis
STI: Sexually Transmitted Infection
MDA: Mass Drug Administration
ARI: Acute Respiratory Infection
UNAIDS: United Nations Acquired Immune Deficiency Syndrome Organization
