# Supplementary material for: One size does not fit all: an application of stochastic modeling to estimating primary healthcare needs in Ethiopia at the sub-national level
Source: BMC Health Serv Res. 2023 Oct 6;23:1070. doi: 10.1186/s12913-023-10061-1 (PMC10559612; doi:10.1186/s12913-023-10061-1)

**Additional file 1**

**Table S1** List of Health Extension Program (HEP) primary health care services(25), grouped in service packages. These services are expected to be delivered at the health posts.

| **Maternal and new-born** |
| --- |
| ANC   - ANC visits - ANC rapid testing   Delivery   - Normal labor management - Active management 3rd stage - Emergency OB - Pre-referral for complications   PNC   - PNC home visits - Maternal sepsis - Mastitis - Postpartum hemorrhage - Pre-referral PPH   Surveillance   - Mortality surveillance - ID fistula and prolapse - Promote cervical cancer ID and treat |
| **New-born and Child Health** |
| - Newborn care - Chlorhexidine - Kangaroo care - Refer congenital anomalies - ICCM childhood illness - IMNCI - Well child check & growth monitoring - Treat severe malnutrition - Treat moderate malnutrition - Deworming & Vitamin A - Pediatric palliative - Pharyngitis, tonsillitis, sinusitis - Scabies - Prevention and identification of child abuse - Pediatric social services |
| **EPI** |
| - Promotion of immunization - Routine immunization - SIAs - RI adverse effects - Outbreak detection & response - Mobilization & promotion of EPI - VPD surveillance |
| **Adolescent and Young Adult Health and Family Planning** |
| - FP counseling - Provision of contraceptives - Provision of implants and IUCD - Removal of implants and IUCD - Psycho-social counseling - Abortion care & referral - Post-abortion follow-up - Education on menstruation - Treatment of menstrual problems - Promote self-breast exam - HPV vaccination - Education on health & wellbeing - Adolescent SRH - Provision of adolescent-friendly contraceptives - Education about GBV - Community dialogue about GBV - Pregnancy test for GBV - HIV test for GBV - Emergency GBV response - Medical treatment for GBV - Psycho-social support for GBV - Advocacy against HTP - Prevention of teen pregnancy - Folic acid - SRH counseling for men |
| **Nutrition** |
| - Promote breast feeding - Nutritional screening during pregnancy - Monthly monitoring - MUAC and oedema screening - Treat malnourished mothers - Anemia - Promote nutrition for MNC - Education re nutrition - Calcium supplementation - Nutrition for HIV-positive women - Promote iodized salt & fortified food - Nutrition outreach - Promote gender equity & empowerment - Nutritional disaster response - Multisectoral nutrition intervention - Breast feeding technique - Acute malnutrition - Counsel complementary feeding - Timely complementary feeding - Promote BF until 24 months - Promote BF during child illness - Educate on complementary foods - Nutrition education for children - Nutrition in schools - Food diversification - School-based de-worming - School nutrition clubs - School gardening programs - School nutrition demonstrations |
| **Major Communicable Diseases** |
| HIV/AIDS and STI   - STI management - STI prevention for sex workers - Risk reduction - HIV self-testing - HIV voluntary counseling & testing - HIV provider-initiated counseling & testing - HIV linkages - Post-exposure prophylaxis - ART refilling/adherence   Malaria   - Vector control - RDT & treat uncomplicated cases - Treat & refer severe cases - Malaria surveillance - Risk reduction - Outbreak detection & response   TB and Leprosy   - Screening for TB & leprosy - Treatment (DOT) - HIV prevention for TB patients |
| **Non-communicable Diseases (NCDs)** |
| Lifestyle   - Promote protection from tobacco smoke - Hazardous alcohol use awareness campaigns - Community-level physical activity   Cancers   - Awareness on risk factors - Screening for common cancers - Breast cancer education - Clinical breast exams - Palliative care for cancer   Hypertension   - Lifestyle counseling - Hypertension screening - Hypertension routine care   Diabetes   - Lifestyle counseling - Screening for type 2 diabetes - Diabetes routine care   Asthma and COPD   - Smoking cessation - Prevention of risk factors - Diagnosis & treat mild asthma - Diagnosis & refer severe asthma   Mental, neurological, and substance use disorders   - Generate community awareness - Identify & link to care - Workplace stress reduction - School-based mental health programs - Identify & assess drug use - Safer storage of pesticides - Emergency poisoning response   Ophthalmic Problems   - Awareness & screening for cataracts - Awareness of eyeglasses - School screenings for vision impairment - Opportunistic screenings for vision impairment - Awareness & screening for glaucoma - Common eye infection treatment   First Aid   - Basic first aid - Advanced first aid   Other Outpatient Services |
| **NTDs** |
| Surveillance for NTDs  Lymphatic filariasis   - LF screening & referral - LF MDA - LLINs and IRS - LF behavior change   Onchocerciasis   - Onchocerciasis MDA - Onchocerciasis behavior change   Trachoma   - Trachoma behavior change - Promote water and sanitation - Dx & treat trachoma - Trachomatous trichiasis surgery - Trachoma MDA - Promote face washing   Schistosomiasis   - Schistosomiasis behavior change - Snail control - Schistosomiasis MDA   Soil Transmitted Helminths (STH)   - STH behavior change - STH MDA - STH dx & treat in pregnancy   Scabies   - Scabies behavior change - Scabies MDA - Diagnosis & treat scabies   Guinea-worm Elimination   - GW vector control - Diagnosis and refer GW - GW behavior change - GW surveillance   Podoconiosis Elimination   - Podo behavior change - Identify & refer infectious lymphedema |
| **Hygiene and Environmental Health** |
| **Health Education and Promotion** |

**Table S2** Probability distribution specifications for stochastic parameters. Random sampling occurs for the listed model parameters based on the chosen probability distribution. Mean and delta values for each model parameter are sourced from reported data. Values for p and q are chosen to reflect the desired range of values to sample from.

| **Model parameter** | **Distribution** | **Inputs for probability distribution** | |
| --- | --- | --- | --- |
| Fertility rates | Uniform | mean,  p = 0.12 | min = mean – mean*p  max = mean + mean*p |
| Mortality rates | Uniform | mean,  p = 0.12 | min = mean – mean*p  max = mean + mean*p |
| Incidence rates | Uniform | mean,  p = 0.1 | min = mean – mean*p  max = mean + mean*p |
| Annual delta fertility rates | Truncated normal | delta,  p = 0.135,  q = 1.35 | mean = delta  std dev = p  a = mean – p*q  b = mean + p*q |
| Annual delta mortality rates | Truncated normal | delta,  p = 0.22,  q = 1.6 | mean = delta  std dev = p  a = mean – p*q  b = mean + p*q |
| Annual delta incidence rates | Truncated normal | delta,  p = 0.1,  q = 2 | mean = delta  std dev = p  a = mean – p*q  b = mean + p*q |
| Minutes per contact | Lognormal | Mean =1,  p = 0.1 | meanlog = log(mean) $-\frac{1}{2}log[\left( \frac{p}{mean} \right)^{2}+1]$  sdlog = $\sqrt[2]{log[\left( \frac{p}{mean} \right)^{2}+1]}$ |

**Table S3** Seasonality Curves. Each column of the table represents one seasonality curve, reflecting the relative frequency of observing the condition in each month of the year. The sum across all months of the year for each curve is 1.

| Month | Malnutrition (21) | TB (23) | Births (19) | Malaria (22) | Diarrhea (20) |
| --- | --- | --- | --- | --- | --- |
| Jan | 0.16 | 0.08 | 0.08 | 0.07 | 0.10 |
| Feb | 0.07 | 0.09 | 0.09 | 0.06 | 0.11 |
| Mar | 0.02 | 0.09 | 0.08 | 0.06 | 0.13 |
| Apr | 0.02 | 0.09 | 0.08 | 0.06 | 0.12 |
| May | 0.03 | 0.09 | 0.11 | 0.06 | 0.10 |
| June | 0.03 | 0.09 | 0.09 | 0.08 | 0.07 |
| July | 0.04 | 0.08 | 0.08 | 0.09 | 0.06 |
| Aug | 0.07 | 0.08 | 0.10 | 0.07 | 0.06 |
| Sept | 0.11 | 0.08 | 0.09 | 0.09 | 0.06 |
| Oct | 0.15 | 0.08 | 0.09 | 0.13 | 0.06 |
| Nov | 0.15 | 0.08 | 0.07 | 0.13 | 0.07 |
| Dec | 0.15 | 0.08 | 0.05 | 0.09 | 0.08 |

**Table S4** Seasonality Offsets. Seasonality curves are applied to relevant clinical tasks based on the listed offset values for each contact. An offset value specifies the number of months a seasonality curve needs to shift to match the timing of the contact with the health system to receive service: 0 for as is, a positive value for shifting forward, and a negative value for shifting backward.

| Clinical Task | Seasonality Curve | Number of Contacts | Offset value for each contact |
| --- | --- | --- | --- |
| ANC visits | Births | 4 | -7, -5, -3, -1 |
| ANC rapid testing | Births | 1 | -4 |
| Normal labor management | Births | 1 | 0 |
| Emergency OB | Births | 1 | 0 |
| Active management 3rd stage | Births | 1 | 0 |
| Newborn care | Births | 2 | 0, 1 |
| Postnatal care | Births | 2 | 1, 2 |
| Maternal sepsis | Births | 1 | 1 |
| RI 1st year | Births | 4 | 4, 6, 9, 12 |
| RI 2nd year | Births | 1 | 15 |
| Well child check & growth monitoring | Births | 3 | 6, 8, 10 |
| Treat malnourished mothers | Malnutrition | 2 | 0, 3 |
| Treat severe malnutrition | Malnutrition | 4 | 0, 1, 2, 3 |
| Treat moderate malnutrition | Malnutrition | 2 | 0, 1 |
| Uncomplicated malaria in adults | Malaria | 1 | 0 |
| Uncomplicated malaria in children | Malaria | 1 | 0 |
| Testing non-malaria cases | Malaria | 1 | 0 |
| Severe malaria in adults | Malaria | 1 | 0 |
| Severe malaria in children | Malaria | 1 | 0 |
| Testing | TB | 1 | 0 |
| Follow-up and linkage for positives | TB | 1 | 0 |
| Diarrhea | Diarrhea | 1 | 0 |

**Figure S1** Population composition in 2020 by age group and region. The total population is shown, broken out by age group, gender, and region. The population includes individuals between the age of 0 and 100, assigned into following groups: infants (i.e., age under 1), under 5 (i.e., age of 1 to 4), 5-14, 15-24, 25-34, 35-44, 45-54, 55-64, 65-100.


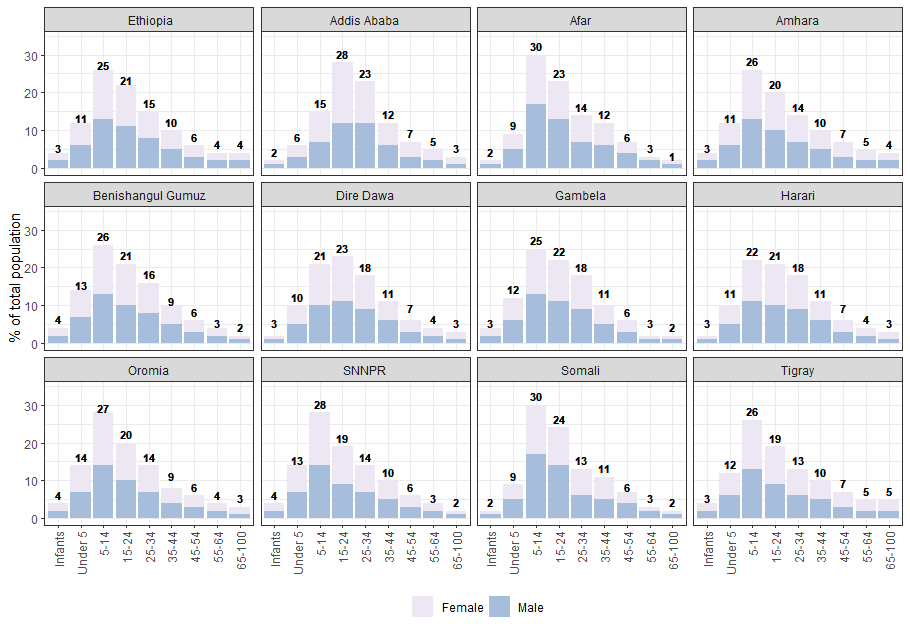


**Figure S2** Seasonality of clinical workload. The month-by-month variation of the clinical workload is shown, broken out for each region, in 2022 and 2035. Values are the ratio of predicted clinical workload for the month, relative to the average predicted monthly workload of the year. Shown are the average values calculated from 100 simulation trials.


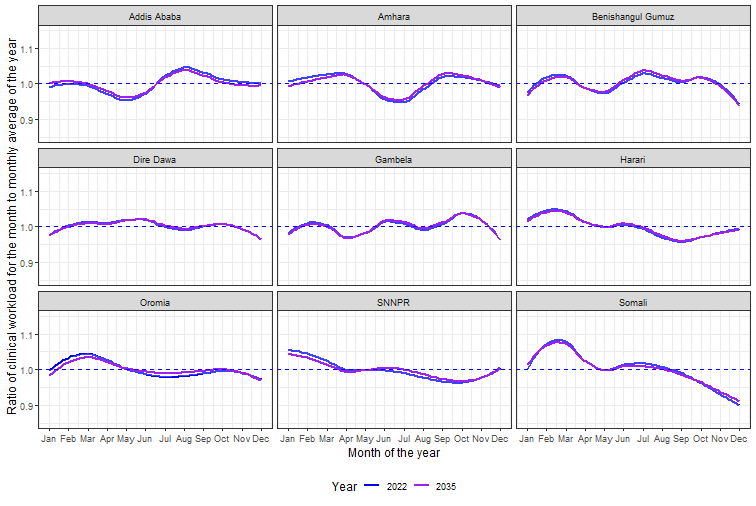

Supplement: Supplementary file 1 — Additional file 1: Table S1. List of Health Extension Program (HEP) primary health care services(25), grouped in service packages. These services are expected to be delivered at the health posts. Table S2. Probability distribution specifications for stochastic parameters. Random sampling occurs for the listed model parameters based on the chosen probability distribution. Mean and delta values for each model parameter are sourced from reported data. Values for p and q are chosen to reflect the desired range of values to sample from. Table S3. Seasonality Curves. Each column of the table represents one seasonality curve, reflecting the relative frequency of observing the condition in each month of the year. The sum across all months of the year for each curve is 1. Table S4. Seasonality Offsets. Seasonality curves are applied to relevant clinical tasks based on the listed offset values for each contact. An offset value specifies the number of months a seasonality curve needs to shift to match the timing of the contact with the health system to receive service: 0 for as is, a positive value for shifting forward, and a negative value for shifting backward. Figure S1. Population composition in 2020 by age group and region. The total population is shown, broken out by age group, gender, and region. The population includes individuals between the age of 0 and 100, assigned into following groups: infants (i.e., age under 1), under 5 (i.e., age of 1 to 4), 5-14, 15-24, 25-34, 35-44, 45-54, 55-64, 65-100. Figure S2. Seasonality of clinical workload. The month-by-month variation of the clinical workload is shown, broken out for each region, in 2022 and 2035. Values are the ratio of predicted clinical workload for the month, relative to the average predicted monthly workload of the year. Shown are the average values calculated from 100 simulation trials. [file 12913_2023_10061_MOESM1_ESM.docx]
